# Drug-Induced Liver Disturbance During the Treatment of COVID-19

**DOI:** 10.3389/fphar.2021.719308

**Published:** 2021-08-18

**Authors:** Guanghua Zhai, Meifen Li, Ying Wang, Jian Wu

**Affiliations:** ^1^ Department of Clinical Laboratory, The Affiliated Suzhou Hospital of Nanjing Medical University, Suzhou Municipal Hospital, Gusu School, Nanjing Medical University, Suzhou, China; ^2^ Department of Infection Management, Suzhou Hosptial Affiliated to Nanjing Medical University, Suzhou, China; ^3^ State Key Laboratory for Diagnosis and Treatment of Infectious Diseases, National Clinical Research Center for Infectious Diseases, The First Affiliated Hospital, Zhejiang University School of Medicine, Hangzhou, China

**Keywords:** severe acute respiratory syndrome coronavirus 2, coronavirus disease 2019, liver disturbance, drugs, treatment

## Abstract

An outbreak of severe acute respiratory syndrome coronavirus 2 (SARS-CoV-2) occurred in Wuhan, China, at the end of 2019. The World Health Organization named the resulting infectious disease as coronavirus disease-2019 (COVID-19). Many studies concluded that patients infected with SARS-CoV-2 have different degrees of liver disturbance. However, the relationship between the drugs used for COVID-19 treatment and liver disturbance remains controversial. It is essential to evaluate the potential liver damage caused by various drugs in order to help guide clinical practice. This review analyzed the effect of drugs on hepatic function during the treatment of COVID-19.

## Introduction

An infectious pneumonia swept across China at the end of 2019. It was confirmed that the pneumonia was caused by severe acute respiratory syndrome coronavirus 2 (SARS-COV-2), and was named as coronavirus disease 2019 (COVID-19) (Wu et al., 2020a). Chai et al. (2020) *.* have shown that SARS-CoV and SARS-CoV-2 bind to angiotensin-converting enzyme two receptors of infected cells, and have found 82% homology between the genome sequence of the novel coronavirus and the chromosome complement sequence of human SARS-CoV (Chai et al., 2020). As many as 60% of SARS patients show abnormal indicators of liver function in investigations of SARS patients (Feng et al., 2020; Morgan et al., 2020). In patients with COVID-19, slightly elevated bilirubin levels and aberrant aspartate aminotransferase (AST) or alanine aminotransferase (ALT) levels are the main manifestations of anomalous liver function (Wu et al., 2020b). Wang *et al.* analyzed the blood biochemical test results of 69 patients (Wang et al., 2020a), and found that 19 patients had elevated AST levels (28%), and 23 patients had elevated ALT levels (33%). In a study of 43 COVID-19 patients, Huang *et al.* found abnormal blood biochemical features, which mainly consisted of higher-than-normal levels of ALT or AST; in addition, they found that one patient developed severe liver injury (Huang et al., 2020a). An investigation of 298 COVID-19 patients reported that 44 patients (14.8%) showed signs of liver damage, and that patients with severe liver disturbance had a higher possibility of deteriorating than did those with mild liver disturbance (Cai et al., 2020). Zhang et al. (2020a) investigated 82 deaths from COVID-19, and found that the incidence of liver disturbance was as high as 78%. Piano et al. (2020) also demonstrated that COVID-19 patients with abnormal liver-function test results had a higher risk of the composite endpoint of death or transfer to an intensive care unit.

In the present review, we comparatively analyzed the pathogenesis of liver disturbance in COVID-19 patients in order to provide a valuable reference for clinical management.

### Hydroxychloroquine and Chloroquine

According to reports, 14.8%–53% of COVID-19 patients have abnormal liver function, including abnormal ALT and gamma-glutamyl transferase levels and hypoproteinemia (Morgan et al., 2020). However, the liver function anomaly in these patients was usually mild to moderate, and severe adverse events typically did not occur. Hydroxychloroquine (HCQ) was initially considered to have antiviral effects and low cytotoxicity in *vitro* experiments (Wang et al., 2020b; Colson et al., 2020). However, several randomized controlled trials and multi-center clinical studies found that HCQ did not increase the negative rate of virus detection or shorten the duration of negative results among COVID-19 patients, and was even associated with more side effects, mainly dizziness and headache, than were observed in patients treated without HCQ (Ip et al., 2020; Mahévas et al., 2020; Ren et al., 2020; Tang et al., 2020). Studies have shown that there is no difference in liver-function abnormalities, including elevated ALT and AST levels, and hypoproteinemia, between COVID-19 patients treated with and without HCQ (Chen et al., 2020).

A systematic review summarized the results of randomized controlled trials of HCQ or chloroquine (CQ) vs general medical care in patients with different viral infections, and compared the safety of HCQ and CQ (Ren et al., 2020). It found that overall, HCQ and CQ were associated with a greater number of side effects than general medical care (relative risk, 1.28 vs 2.25). However, the study also found that the HCQ/CQ group did not have significantly more severe side effects than the general care group. Liver dysfunction in both group was classified as a mild adverse event. Therefore, in general, HCQ did not cause severe side effects in COVID-19 patients. This systematic review provides a basis for drug safety in the application of HCQ/CQ to the management of COVID-19.

Owing to the *in vitro* antiviral effect of HCQ/CQ mentioned above, HCQ/CQ began to be gradually applied to COVID-19 treatment. After several clinical trials, a meta-analysis of three randomized controlled clinical trials reported that in two of the clinical trials, the side effects, including liver-function abnormalities, were not significantly different between the HCQ/CQ and control groups (Amani et al., 2020). In another study, which included a total of 150 people, the final conclusion was that the HCQ group had more side effects than the non-HCQ group; moreover, the side effects included both severe adverse events, such as disease progression and acute respiratory distress, and mild events, such as nausea, vomiting, abdominal discomfort, and mild liver function abnormality (Tang et al., 2020). Although the total number of side effects was greater in the HCQ group than in the non-HCQ group, the number of patients with liver function abnormality did not differ between the two groups; in fact, no patient in the HCQ group developed abnormal liver function, and one patient in the non-HCQ group developed abnormal liver function. This suggests that in COVID-19 patients treated with HCQ, the occurrence of abnormal liver function is not common, and HCQ may not be the cause of liver disturbance in COVID-19 patients. Furthermore, the REFHEPS, a European French-speaking study network focused on drug-induced liver disturbance, showed that HCQ is a possible but rare cause of idiosyncratic drug-induced liver disturbance (Olry et al., 2020).

### Azithromycin

Azithromycin is a macrolide antibiotic that was widely used to treat bacterial infections before it was applied to the treatment of COVID-19 (Bermejo-Martin et al., 2009; Min and Jang, 2012; Lin et al., 2016). Some studies concluded that this drug may cause acute cholestatic hepatitis within 1–3 weeks after starting treatment (LiverTox). A team from Japan evaluated the safety of azithromycin in patients with mild or moderate community-acquired pneumonia (Noguchi et al., 2014). They found that although the efficacy of azithromycin seemed good, with 88.5% of patients showing clinical recovery, the associated liver dysfunction could not be ignored; of 62 patients, 15 developed liver dysfunction by the end of the follow-up period. Thus, a rigorous evaluation of the safety of azithromycin in patients with COVID-19 is urgently needed. Azithromycin has been suggested to improve virus clearance and clinical outcomes, though certain benefit has not been fully confirmed.

Studies on azithromycin-induced liver damage in patients with COVID-19 are limited. However, according to a multinational cohort study, azithromycin had a similar hepatic failure rate to amoxicillin, which has low hepatotoxicity (Lane et al., 2020). One open-label observational study reported that the combination of azithromycin with HCQ may lead to a faster recovery and reduction of virus load in the treatment of COVID-19 (Hulme et al., 2021). Nevertheless, many researchers do not agree with this conclusion owing to its limited evidence power and shortcomings in the study design. Moreover, as azithromycin is known to cause liver disturbance in patients with bacterial infections, it indeed could cause liver injury in patients with COVID-19. However, we should keep in mind that drug safety is determined by not only the drug characteristics but also the type of infection. More detailed clinical trials are required to determine the association between azithromycin and liver disturbance in patients with COVID-19.

In studies about azithromycin, the incidence of liver injury was low, usually less than 10%. Thus, considering that 14.8–53% of SARS-CoV-2 patients are reported to show indicators of liver disturbance (mainly elevated ALT, hypoalbuminemia, and elevated AST), the incidence of liver disturbance in patients treated with azithromycin is low (Jeronimo et al., 2020).

### Lopinavir and Ritonavir

In a randomized controlled study on lopinavir/ritonavir (LPV/r)-treated adult patients hospitalized with mild/moderate COVID-19, only one patient in the LPV/r group developed elevation of ALT over 2.5-fold above the normal limit (Li et al., 2020). In contrast, none of patients in the arbidol (control) group developed liver dysfunction. Furthermore, the patient with ALT elevation had a pre-existing chronic liver condition, which could have contributed to the liver disturbance. In addition, during follow-up, no evidence was found indicating that LPV/r could exacerbate a mild/moderate clinical status to a severe/critical clinical status. Finally, there were no deaths, either in the LPV/r group or arbidol group.

According to another research study, no apparent side effects were found in the LPV/r group, except for transient ALT elevation (<125 U/L) in three patients in the first week of admission (Zhu et al., 2020). Considering that none of the patients progressed to severe/critical clinical status, and that all patients were alive at the end of the follow-up period, we believe that LPV/r treatment rarely causes harm in patients recovering from COVID-19.

In a small sample of 22 patients, 12 patients in the LPV/r group displayed normal levels of ALT (median: 18.55 U/L) and AST (median: 19.00 U/L), and AST/ALT elevation did not develop in any patient until the end of the follow-up period (Naksuk et al., 2020). Although the sample size was small, this study showed that the incidence of liver disturbance in patients treated with LPV/r was low. As for the clinical outcomes, the proportion of negative RT-PCR results on day 14 was 91.67 and 100% in the LPV/r group and CQ group, respectively.

Another study investigated the efficiency of LPV/r therapy in reducing the abnormal values of the liver biochemical indexes ALT and AST, and showed a lower incidence of ALT and AST elevation in the LPV/r group than in patients treated with adjuvant drugs only (Ye et al., 2020). At the standard dose of 400/100 mg, it seems that LPV/r does not impair liver function.

One study evaluated the safety of LPV/r in a large sample of 199 patients (Cao et al., 2020). The authors reported that although the incidence of pre-existing liver conditions was similar in the LPV/r and control groups, fewer patients in the LPV/r group than in the control group exhibited ALT dysfunction during treatment, though the difference was not statistically significant. Moreover, given that the mortality rate was lower in the LPV/r group than in the control group, the LPV/r-treated patients with ALT elevation may not be hurt by this therapy.

Given the low incidence of liver disturbance, which mainly consisted of elevated ALT or AST levels, in comparison with the control group, we found no evidence that LPV/r causes liver disturbance in patients with COVID-19. Moreover, during a usual follow-up of 14 days, the proportion of patients in whom a mild/moderate clinical status changed to a severe/critical clinical status was similar in the LPV/r and control groups. All studies, except one, used a dose of 400/100 mg for the LPV/r treatment. More clinical trials on the dose of LPV/r should be performed to further evaluate the safety of this treatment.

### Remdesivir

Remdesivir is a monophosphoramidate prodrug of an adenosine analogue with a broad antiviral spectrum that includes coronaviruses (Warren et al., 2016; Lo et al., 2017; Sheahan et al., 2017; Pardo et al., 2020). Several studies have found an antiviral effect of remdesivir on SARS-CoV-2, in both *in vitro* and animal models (Zhang et al., 2020a). However, its clinical benefit has not been confirmed due to limited sample sizes and questions regarding the safety of remdesivir. Contradictory conclusions have been reported in several clinical trials. Safety is the main factor determining the effectiveness of remdesivir. The association of remdesivir with liver injury remains uncertain, and is essential to evaluate the safety of this drug. One randomized multicenter trial enrolled 237 patients with COVID-19 (Wang et al., 2020c), and found similar ALT and AST levels between the remdesivir and placebo groups; the final proportion of patients with increased AST levels did not differ between these two groups. In fact, 5% of the patients in the remdesivir group had elevated AST, while 12% of those in the placebo group had elevated AST. Furthermore, the mortality rates did not differ between these two groups on days 7 and 14. This suggests that remdesivir is not a major cause of liver disturbance in patients with COVID-19.

In another large-scale clinical research study with 1,063 patients, although the efficiency of remdesivir against COVID-19 was opposite to that reported by the above study, the trend of liver transaminases remained the same (Beigel et al., 2020; Davies et al., 2020). The proportion of patients with elevated AST levels was 2.8% in the remdesivir group and 3.8% in the placebo group; the proportions of patients with increased ALT were 1.5 and 1.7% in the remdesivir and placebo groups, respectively. Compared to the placebo, remdesivir showed a trend of protection from liver disturbance, though the results were not statistically different. Consistent with this, the mortality rate was significantly lower in the remdesivir group than in the placebo group, especially among patients with a severe clinical status.

The effect of a drug may be time- and dose-dependent. Usually, remdesivir was used throughout the clinical course or until the occurrence of severe adverse events. However, it remains unknown whether remdesivir can be administered for a long duration. One randomized phase-3 trial evaluated the safety of continuous remdesivir usage (Goldman et al., 2020). Patients were administered 200 mg remdesivir on day 1 and 100 mg once daily thereafter for either five or 10 days. The study found no significant differences between the 5 days and 10 days groups after adjustment for baseline clinical status. Importantly, 11 of the 200 patients in the 5 days group and eight of the 197 patients in the 10 days group showed increased ALT levels. A similar trend was observed for AST elevation; 5 and 7% of patients showed mild AST elevation in the 5 days and 10 days groups, respectively. The authors concluded that liver disturbance was rare in both groups, and that there was no statistical difference between the two groups. Thus, considering the duration of treatment and comparison with the placebo, we believe that the association between remdesivir and liver injury in patients with COVID-19 was weak (Roshanshad et al., 2020).

Although some large sample review analysis concluded that remdesivir may not affect the liver function of patients, we still cannot take it lightly based on the pharmacological characteristics of remdesivir. For example, five patients infected with SARS-CoV-2 from Italy revealed various liver function changes during hospitalization. Three of them showed significant increase in ALT or AST when switched to remdesivir (Zampino et al., 2020). Therefore, we should be cautious about the use of remdesivir.

Although most clinical trials of remdesivir have not shown a significant association between treatment and liver toxicity, some small-scale trials have reported that remdesivir can cause an increase in ALT/AST, so remdesivir should be used with close monitoring of liver function tests, and patients with prior liver diseases should receive intensive care. More lager randomized controlled trials with powerful evidence should be carried out to confirm these results.

### Interferon

Interferon (IFN) has been used for the treatment of SARS and Middle East respiratory syndrome (MERS), and has shown antiviral effects, especially, IFN-β and IFN-γ on SARS and MERS-CoV (Scheuplein et al., 2015; Jeon et al., 2020). Although IFNs have been used in some patients with COVID-19, their efficacy in the treatment of COVID-19 remains to be proved. Furthermore, the safety of IFNs should also be evaluated. One clinical trial has assessed the efficacy and safety of IFN in patients with severe COVID-19 (Davoudi-Monfared et al., 2020). In this trial, patients were injected with IFN-β-1a 3 times a week for 2 weeks, and the 28 days mortality and hepatic complication rates were measured and compared with patients receiving standard care. Initially, the characteristics of the IFN and control groups were well balanced. The mean AST level was 50 U/L in both the IFN and control groups, while the mean ALT level was 44 U/L and 41 U/L in the IFN and control groups, respectively. The incidence of pre-existing liver disease was also balanced. The incidence of hepatic failure did not differ between the IFN and control groups (11.90 vs 23.07%, respectively), suggesting that IFN-β-1a may not play a major role in liver injury in COVID-19 patients. The 28 days mortality was also lower in the IFN group than in the control group, indicating that IFN-β-1a may be effective for the treatment of COVID-19.

IFN-β-1b has been found to reduce the viral replication rate in Vero E6 cells infected with SARS-CoV-2. In a retrospective clinical study, comorbidities were compared between the IFN and control groups (Estebanez et al., 2020). The number of comorbidities (either one or two comorbidities per patient) was the same in both groups. The main comorbidities were hypertension, dyslipidemia, and cardiopathy; abnormal liver enzymes were rare. None of the patients in the IFN group withdrew from the study due to liver disturbance.

Overall, IFNs, including IFN-β-1a and IFN-β-1b, were unlikely to be associated with liver disturbance in patients with COVID-19, although these drugs may lead to hepatic toxicity when used together with other drugs. More detailed clinical trials are needed to confirm these results.

### Traditional Medicine

Traditional Chinese medicine (TCM) formulate treatment based on symptom-based diagnosis, an approach which is increasingly emphasized in other disciplines (Chan et al., 2020). For example, for treating mild case named “Cold-dampness distressing lung”, recommended drugs include Ma-huang 6 g, shi-gao 15 g, xing-ren 9 g, qiang-huo 15 g, ting-li-zi 15 g, guan-zhong 9 g, di-long 15 g, xu-chang-qing 15 g, huo-xiang 15 g, pei-lan 9 g, cang-zhu 15 g, fu-ling 45 g, bai-zhu 30 g, shan-zha 9 g, shen-qu 9 g, mai-ya 9 g, hou-pu 15 g, bin-lang 9 g, cao-guo 9 g, sheng-jiang 15 g according to Chinese National Guideline. A cohort study enrolled 135 COVID-19 patients and 91.8% of the patients were treated with traditional Chinese medicine. Finally, liver disturbance was not found till the end in the whole cohort (Wan et al., 2020). Similarly, another retrospective study was performed in China and 273 out of 293 patients were treated with traditional Chinese medicine. Liver function remained the novel level in both moderate and severe groups (Shu et al., 2020). This is consistent with other cohort studies (An et al., 2021; Wang et al., 2021). The Indian traditional medical system is one of the oldest medical practice systems in the world. India has its own recognized unique distinctions in traditional medicine including Ayurveda, Yoga, Unani, Siddha and Homeopathy (AYUSH) (Ahmad et al., 2020). RCT study on AYUSH are not enough and effect of AYUSH on liver function remains uncertain.

### Other Drugs

Other drugs that have been used for the treatment of patients with COVID-19 include baricitinib, imatinib, and darunavir (Bernal-Bello et al., 2020; Richardson et al., 2020). These drugs are still in the experimental stage, and have not been widely applied in clinical practice; thus, their potential to cause liver disturbance in COVID-19 patients remains unknown.

### The Mechanisms of Drug-Induced Liver Disturbance in the Treatment of COVID-19

The mechanism of drug-induced liver disturbance in the treatment of COVID-19 has also been reported. It is well known that nucleoside analogues are an important class of antiviral drugs, which can cause liver disturbance through a variety of mechanisms. The most typical mechanism is a mitochondrial type of liver disturbance, which may be due to the incorporation of nucleoside analogues into or blocking mitochondrial DNA synthesis by mitochondrial gamma polymerase, leading to mitochondrial depletion or functional decline. Mitochondrial damage can affect multiple tissues, leading to myopathy, neuropathy, pancreatitis, myelosuppression and liver disturbance (Lu, 2020; National Institute of Diabetes and Digestive and Kidney Diseases, 2012). Huang C *et al.* showed that severe sepsis patients with COVID-19 may have hypoxic liver disturbance caused by ischemia and shock, cholestasis related liver disturbance caused by bile metabolism changes, and hepatocyte injury caused by drug toxicity or excessive inflammation, which suggested that the toxicity of antiviral drugs and hypoxia reperfusion may be related to liver disturbance in patients with COVID-19 (Huang et al., 2020b). Ferron PJ *et al.* also found that some antiviral drugs including corticoids, antiretroviral agents and methotrexate may trigger the transformation of fatty liver to NASH, or aggravate the existing steatosis, necrotizing inflammation and fibrosis in patients with COVID-19. The mechanisms include the impairment of exogenous metabolic enzyme activity, mitochondrial dysfunction, changes in lipid homeostasis and oxidative stress (Ferron et al., 2020).

### Pre-Existing Liver Diseases

Patients with pre-existing liver diseases often have liver dysfunction (Garrido et al., 2020). As the elevated ALT and AST were preciously supposed to be associated with the severity of COVID-19, whether these chronic liver diseases affect the prognosis remains uncertain. This may help manage the treatment strategy of these patients. Thus, till now, several observational studies have mentioned the distributions of liver biochemicals in different degrees of patients with COVID-19 (Guan et al., 2020).

### Chronic Liver Disease

A comprehensive analysis of patients from Zhejiang province in China was performed, with a total of 465 patients (Lian et al., 2020). In the baseline characteristics, chronic liver disease (CLD) accounted for 4.09% of the whole patients and the increased ALT and AST were 10.11 and 11.18%, respectively. Also, the incidence of increased total bilirubin was 27.96%. Considering that the antiviral drugs have not been applied, drug-induced liver injury shall not be the main reason for liver dysfunction in patients with COVID-19. During the treatment, 61 out of 465 patients suffered liver injury which was mild higher than that at the baseline, indicating that CLD may result in the initial liver dysfunction but was not associated with elevated ALT or AST during the course of treatment. As many research found that elevated ALT or AST was different between mild/moderate COVID-19 and severe/critical COVID-19 and abnormal liver tests were associated with worse prognosis. In this study, the increased ALT and AST were two risk factors for the severe of patients (both *p* < 0.05). However, by multivariate analysis of risk factors of severe COVID-19 patients, pre-existing liver disease was not associated with the severe of COVID-19.

Another study mentioned CLD in patients from Wuhan, China (Xu et al., 2020). The baseline coexisting conditions were similar with 4 (12%) liver diseases in mild group while 3 (10%) in severe group. And baseline increased AST was balanced in two groups as excepted. At baseline, the elevated AST was 5 (15%) in mild group while 5 (17%) in severe group. It could be guessed that the liver dysfunction was likely due to the previous liver diseases. From another aspect, as these patients were not taken drugs at admission, the initial liver injury could not be the result of antiviral drugs. Moreover, the study found that patients with previous CLD did not increase the risk of disease progression or mortality.

The two trials above considering CLD status between different degrees of COVID-19 showed that although the previous CLD may not induce the COVID-19 to progress, the liver injury at baseline shall result from previous CLD. This give an explanation to liver injury during courses of COVID-19.

There also two studies comparing the characteristics of patients with or without ICU care (Huang et al., 2020a; Wang et al., 2020d). Patients with ICU care because they needed high-flow nasal cannula or higher-level oxygen support to correct hypoxaemia, which mainly meets the criteria of severe/critical COVID-19. In a cohort from Wuhan, China, the proportion of CLD was small while the increased ALT was rare. And owing to the high incidence of pre-existing cardiovascular disease, 25% in ICU group and 10.8% in non-ICU group, the number of increased AST was relatively larger than that of ALT.

However, according to a large cohort in England, the previous liver disease was associated with higher risk of COVID-19 hospital death adjusted by age and sex (Williamson et al., 2020). Considering it was a large-scale cohort, the selective bias would be small. In overall, it may be the result of heterogeneity in CLD.

This was consistence with the study carried by another team from China (Cai et al., 2020). In overall, the proportion of previous CLD and the proportion of liver enzymes, especially ALT, have a similar trend, which means liver injury in patients with COVID-19 may be caused by pre-existing liver diseases. Nevertheless, CLD contains several kinds of liver diseases, which may have different effect on liver injury. It is essential to discuss separately.

### Chronic Hepatitis B

Hepatitis B virus (HBV) is one of the viruses that causes a worldwide infection and thus, became a globe problem (Ott et al., 2012). The incidence of HBV infection is high in China and may cause liver injury in COVID-19. However, researches on effect of HBV on patients during the course of COVID-19 infection are limited. A retrospective investigation showed that the median ALT was 25.0 (16.0–44.0) and 21.5 (15.0–32.8) in patients with and without group, respectively; the median AST was 28.0 (19.0–58.0) and 25.0 (19.0–37.0), respectively (Zou et al., 2020). But the total bilirubin was significantly higher in patients with HBV infection. Although the proportion of elevated ALT or AST was not displayed and current levels of AST and ALT were not significantly different, total bilirubin could prove the liver injury caused by HBV. However, due to the limited sample size of patients with HBV infection, the evidence was not powerful enough. And considering that the replication and liver impairment of HBV could be different at different time points in different phases, more coinfection cases is needed to provide further evidences. For mortality, patients with HBV infection showed higher mortality rate compared with those without HBV infection.

### Non-Alcoholic Fatty Liver Disease

According to several trials which contained pre-existing liver disease, the incidence of abnormal of ALT was between 13.3 and 31.6%. However, patients with non-alcoholic fatty liver disease (NAFLD) in two studies had a much higher incidence of elevated ALT, up to 50% (Zhang et al., 2020b; Ji et al., 2020; Roca-Fernandez et al., 2020). Also, the incidence of elevated gamma-glutamyl transferase (GGT) was 22.8%, which was higher than patients without NAFLD. As one of those was a study with 202 patients, the evidence could be powerful, though more studies on NAFLD should be carried out.

As for the effect of NAFLD on COVID-19, patients with NAFLD often were combined with other coexisting diseases, mainly diabetes, hypertension and obesity, which could cause the severity of COVID-19. Moreover, patients with NAFLD had a higher risk of COVID-19 progression. Although NAFLD provides the pathological fundamentals to liver injury and the trend of elevated ALT and GGT was also consistent with what we supposed, more confident results are warranted. More importantly, considering the possible liver injury, patients with NAFLD should be taken protective measures to prevent from critical outcomes.

## Conclusions and Perspectives

**FIGURE 1:**
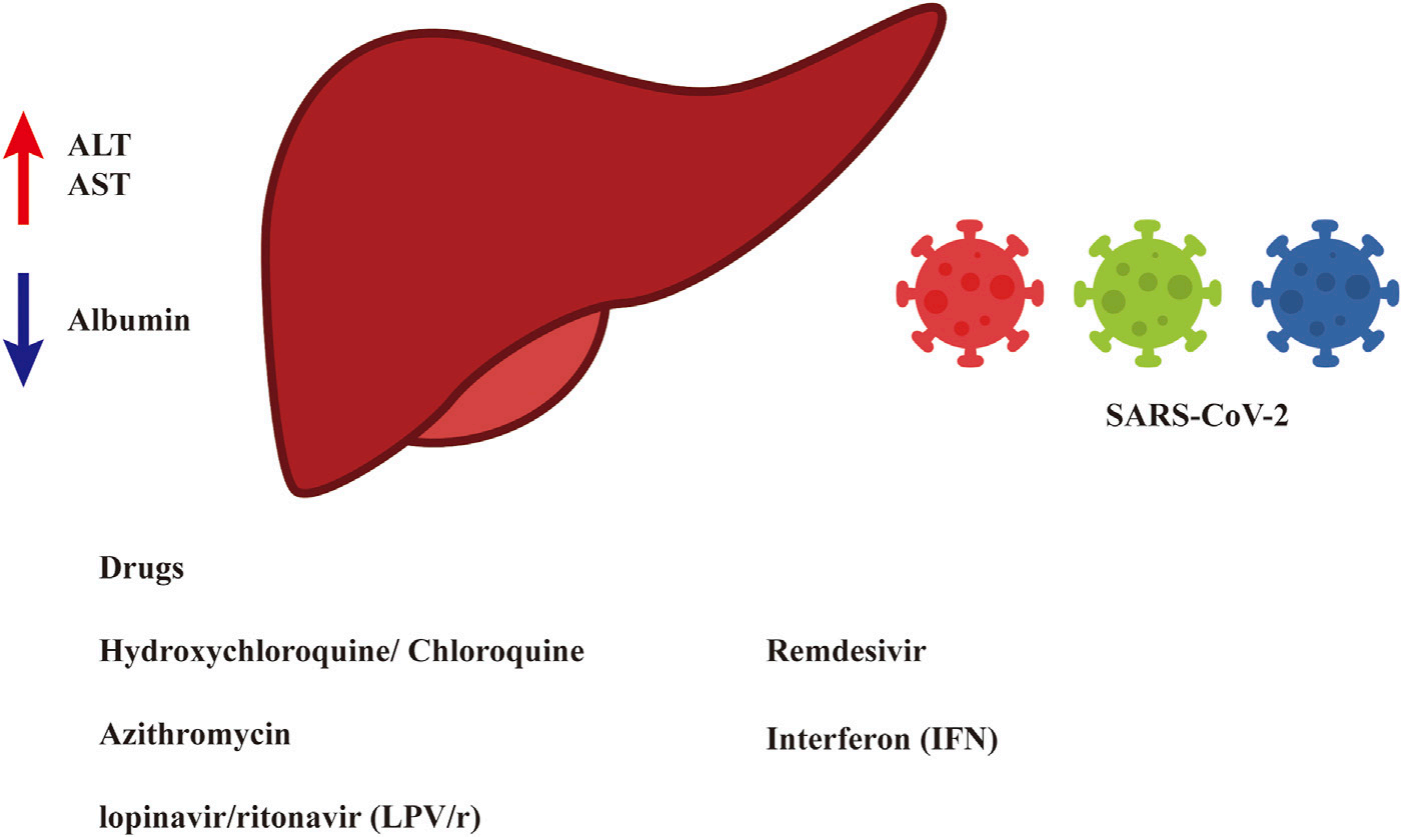
Liver disturbance during the treatment of COVID-19.

Several drugs have been widely used for the treatment of patients with COVID-19 and have shown hepatic toxicity when used for patients infected with other viruses. This review evaluated the use of HCQ/CQ, azithromycin, LPV/r, remdesivir, IFN, and some other antiviral drugs in the management of COVID-19 patients (Table 1). Considering that these treatments were associated with minimal liver function abnormalities, specifically liver-enzyme elevations, and tended to decrease the mortality rate of COVID-19 patients, we have shown that there is low-level evidence that the above drugs cause liver disturbance. However, the risk of liver disturbance cannot be ignored altogether, as in clinical practice, multiple drugs are often administered together. Although we may assume that liver disturbance was not mainly caused by any one drug in several clinical trials, it is possible that the combination of multiple drugs may lead to liver disturbance (Figure 1). For example, in the evaluation of the efficacy of IFN-β-1a as compared to a control treatment, the patients in the control group received standard care that included HCQ and LPV/r. The incidence of hepatic failure was 11.90 and 23.07% in the IFN and control groups, respectively, which was a result of the combination of IFN and other drugs. Thus, more research is needed.

**TABLE 1:** Characteristics of the included studies.

Authors	Study type	Country	Sample size	Drug	Outcomes
Chen et al. (2020)	RCT	China	62	HCQ	No liver injury was found in COVID-19 patients
Ren et al. (2020)	Meta-analysis	\	2,137	HCQ/CQ	The incidence of liver damage was 0.5% in the HCQ group and 0.2% in the placebo group
Tang et al. (2020)	RCT	China	150	HCQ	No patient had abnormal liver function in the HCQ group, and 1 patient had abnormal liver function in the general medical care group
Li et al. (2020)	RCT	China	86	LPV/r	One patient developed elevation of ALT over 2.5-fold above the normal limit in the LPV/r group
Zhu et al. (2020)	Cohort	China	50	LPV/r	Transient ALT rise (<125 U/L) in 3 patients in the LPV/r group
Naksuk et al. (2020)	Review	\	22	LPV/r	No elevated AST or ALT in any patient
Cao et al. (2020)	RCT	China	199	LPV/r	Fewer patients exhibited abnormal ALT levels during treatment in the LPV/r group vs the control group
Wang et al. (2021)	RCT	China	237	Remdesivir	The rate of increased AST did not differ between the remdesivir and control groups
Beigel et al. (2020)	RCT	United States	1,063	Remdesivir	The proportion of patients with increased AST was 2.8% in the remdesivir group and 3.8% in the placebo group
Goldman et al. (2020)	RCT	United States	397	Remdesivir	Mild AST elevation occurred in 5% of patients in the 5-days group and 7% of patients in the 10-days group
Davoudi-Monfared et al. (2020)	RCT	Iran	92	IFN	The mean AST level was the same (50 U/L) in the IFN and control groups
Estebanez et al. (2020)	Cohort	Spain	256	IFN	The incidence of abnormal liver enzymes was the same in both study groups

Based on the current evidence, the trend of liver disturbance caused by antiviral drugs used to treat COVID-19 patients is not obvious.
